# Dermoid Cyst of the Ovary

**Published:** 1876-07

**Authors:** L. P. Rosser

**Affiliations:** Conyers, Ga.


### DERMOID CYST OF THE OVARY.

By L. P. ROSSER, M.D., of Conyers, Ga.

Mrs. V., a married woman, thirty-five years of age, of a healthy constitution, who had previously borne one child; some years before was supposed to be pregnant, and at the end of her second gestation was seized with pains resembling the pains in natural labor. The family physician was called in, and remained with her some twenty-four hours, at the end of which time the pains ceased entirely, and did not return again. The family physician informed me that she had all the signs of pregnancy up to this attack.

Mrs. V. survived some seven years, and during which time her health was bad, though she was never confined to her bed until a short time before her death. She had a gradual enlargment of the abdomen during these seven years of her life, until at death she was of an enormous size. I got the permission of her husband and family to perform a post mortem, and found the right ovary enlarged to about the size of an ordinary water-bucket. I did not weigh the tumor, but suppose it would have weighed some twenty pounds or more. The tumor appeared to consist of a large number of cysts, some of them filled with a yellowish watery fluid, and some filled with a thick, yellow, tenacious matter, mixed with nice fine hair, from two to four inches in length, in appearance resembling a child's hair at four or five years of age. The tumor had encroached so much on the Fallopian tube and ligaments that they were lost in the tumor until found by dissection. In tracing the Fallopian tube, I found a hard substance in the cavity of the tube, and on making an incision found two well-formed teeth, one with the fang attached to and imbedded in the muscular coats of the tube; the other one was not attached, but both appeared to be in a perfect state of preservation. The uterus was about the usual size of those found in women who have borne children. I examined the cavity and found nothing unusual connected with that organ. I also learned that this patient had been several times tapped for ascites, without effect.

